# Understanding the microbiome–crop rotation nexus in karst agricultural systems: insights from Southwestern China

**DOI:** 10.3389/fmicb.2025.1503636

**Published:** 2025-02-26

**Authors:** Bin Wang, Nianjie Shang, Xinwei Feng, Zongling Hu, Pengfei Li, Yi Chen, Binbin Hu, Mengjiao Ding, Junju Xu

**Affiliations:** ^1^College of Agronomy and Biotechnology, Yunnan Agricultural University, Kunming, China; ^2^Yunnan Tobacco Company Wenshan Prefecture Company, Wenshan Zhuang and Miao Autonomous Prefecture, Yunnan, China; ^3^Institute of Crop Germplasm Resources, Guizhou Academy of Agricultural Sciences, Guiyang, China; ^4^Guizhou Tobacco Company Qiannan Company, Duyun, China; ^5^Yunnan Academy of Tobacco Agricultural Sciences, Kunming, China; ^6^College of Tobacco Science, Guizhou University, Guiyang, China; ^7^Guizhou Provincial Key Laboratory for Tobacco Quality, College of Tobacco Science, Guizhou University, Guiyang, China; ^8^Zhengzhou Tobacco Research Institute of CNTC, Zhengzhou, China

**Keywords:** crop rotation, soil microbial communities, karst agricultural system, soil physicochemical properties, microbiome assembly

## Abstract

Understanding how soil properties and microbial communities respond to crop rotation is essential for the sustainability of agroecosystems. However, there has been limited research on how crop rotation alters below-ground microbial communities in soils with serious bacterial wilt within the karst agricultural system. This study investigated the effects of continuous planting of corn, tobacco, and tobacco–corn rotation on soil microbial communities in the karst regions of Southwestern China. High-throughput sequencing was used to evaluate the responses of the soil microbial community structure to crop monoculture and rotation patterns. As expected, the tobacco–corn rotation mitigated the negative effects of continuous cropping and reduced soil acidification. The tobacco–corn rotation also significantly altered the composition of microbial communities and promoted plant growth by fostering a higher abundance of beneficial microorganisms. The predominant bacteria genera *Sphingomonas* and *Gaiella* and the predominant fungal genera *Mortierella* and *Saitozyma* were identified as discriminant biomarkers that are critical to soil ecosystem health. pH, available potassium (AK), and available phosphorus (AP) were the primary soil factors related to the soil microbiome assembly. This study aimed to demonstrate the association between crop rotation and microbiomes, suggesting that altering cultivation patterns could enhance karst agricultural systems.

## Highlights

- Corn rotation increased soil bacterial diversity at the Operational Taxonomic Unit (OTU) level.
- Discriminant biomarkers were critical to soil ecosystem health.
- Corn rotation enriched the growth of beneficial microorganisms in the soil.

## Introduction

With the excessive pursuit of economic interests and the diminishing availability of land resources, continuous cropping patterns have become an important part of agricultural production and are widely used in China. However, this practice has led to soil nutrient imbalances and a rise in soil-borne diseases (Machado et al., 2007; Zhang M. M. et al., 2023). Previous reports have indicated that crops have strong interactions with soil, and continuous cropping of the same plant causes root rot (Duddigan et al., 2021; Chen et al., 2022). In monocropping tobacco systems, the most prevalent diseases include bacterial wilt caused by *Ralstonia solanacearum*, root rot caused by *Fusarium oxysporum*, and black shank caused by *Pythium ultimum* (Lee et al., 2021). Certain diseases such as stem rot, ear rot, and common rust caused by *Puccinia sorghi*, *Fusarium subglutinans*, and *Pythium aphanidermatum*, respectively, have become more prevalent due to the continuous cultivation of corn (Liu et al., 2022). Numerous studies have documented that the decrease in crop yield is associated with soil degradation under continuous cultivation (Fujisao et al., 2020; Arunrat et al., 2023). The composition and diversity of microbial communities in soil are critical for maintaining soil health and quality (Bai et al., 2019). Several reports have revealed that imbalances in soil microbial communities correlate with continuous cropping (Fang et al., 2018; Ding et al., 2024). Furthermore, continuous cropping has also been reported to increase the prevalence of harmful fungi (Gao et al., 2021). The ecological imbalance of microbial communities in the rhizosphere soil of plant hosts is an important mechanism for the development of plant diseases. Monoculture crop systems disrupt the soil microbial community and are associated with the lowest levels of microbial diversity (Liu et al., 2021b). The relative abundance of pathogenic fungi was found to increase synergistically with the duration of continuous tobacco cultivation (Ding et al., 2024). Furthermore, the abundance of soil-borne pathogens (e.g., *Fusarium*) increased significantly after cucumbers were monocropped (Jin et al., 2019). The outbreak of bacterial wilt in tomatoes was attributed to the disruption of Firmicutes and Actinobacteria in the tomato rhizosphere (Lee et al., 2021). Therefore, protecting the soil micro-ecosystem is crucial for the healthy growth of crops. However, how does the continuous cropping system change the physical and biochemical properties of soils? How is bacterial wilt caused by the imbalance in the microbial structure within soil? Currently, no definite answers to these questions are available yet.

Rotation patterns alter soil physical and biochemical properties and shift the community structure (McDaniel et al., 2014; Zhang H. F. et al., 2023). The practice of crop rotation is widely adopted for its manifold advantages, which include pest control, disease control, and the enhancement of crop yields (Behnke et al., 2021). Alkali-hydrolyzed nitrogen (AN) and available phosphorus (AP) were increased by paddy upland rotation in one study (Turmuktini et al., 2012). Compared to continuous cropping systems, rotations promote more efficient nutrient cycling (Town et al., 2022). Numerous studies have found that crop rotation increases the number of bacteria and actinomycetes, improves the ratio of bacteria to fungi, and enhances the community structure in soil (Zhang H. F. et al., 2023; Yan et al., 2024). Crop rotation alters the quantity and quality of plant residues, which serve as an energy source for soil microorganisms, leading to changes in the soil microbial community structure (Behnke et al., 2021). The bacterial community richness and Shannon index were higher in tobacco rotation systems compared to continuous cropping systems (Zheng et al., 2020). Tobacco–corn rotation has also been shown to suppress the incidences of viral diseases (Niu et al., 2016). In addition, diverse crop rotations help reduce pathogen host-plant incidence (Floc'h et al., 2020; Xie et al., 2022). However, some problems caused by crop rotation patterns cannot be ignored. For example, soil deterioration and reduced fertilizer utilization have been observed in rice-wheat crop rotation systems (Zhou et al., 2014). The abundance of plant pathogens has been reported to increase in pea rotation systems (Niu et al., 2018). In addition, phytopathogenesis results from interactions between microbiomes associated with plant hosts and pathogens, which play a central role in regulating plant health and pathogen infection (Guo et al., 2024). Therefore, a thorough understanding of changes in the microbial community structure under crop rotation patterns is important for the karst agricultural system.

Wenshan Zhuang and Miao Autonomous Prefecture (Wenshan Prefecture) has a unique geographical location, situated in the Yungui Plateau in Southwestern China, with a typical karst landform. Wenshan Prefecture enjoys sufficient sunshine, with an average annual temperature of 16–19°C and an annual rainfall of 1,075 mm, making it suitable for the growth of various crops (Feng et al., 2018). However, mountainous areas account for 97% of the total land area, with karst regions making up 53.4% (Li and Lu, 2019). The scarcity of land resources and continuous cropping obstacles have led to the outbreak of soil-borne diseases, which have seriously affected the development of industries such as grain, *Panax pseudoginseng*, tobacco, and chili. Crop rotation, as an effective method for preventing and controlling soil-borne diseases, is increasingly being recognized for its role in regulating soil microorganisms. By the time of writing, numerous studies have been conducted on the issue of continuous cropping in soil. However, limited research has elucidated the response of soil microbial communities to crop rotation in soils severely affected by bacterial wilt. Therefore, we employed high-throughput sequencing technology to assess the long-term adaptive differences in soil microbiome characteristics between corn and tobacco fields under monoculture and tobacco–corn rotation. The present study will help understand the effects of crop rotation on the complex interactions between host plants and soil microbial species, including soil health indicators and discriminant biomarkers. We aim to provide a theoretical basis for mitigating soil-borne diseases caused by continuous cropping barriers. The present study also attempted to explore suitable local crop rotation patterns, which are crucial for guiding agricultural practices.

## Materials and methods

### Site description and experimental design

**Figure 1:**
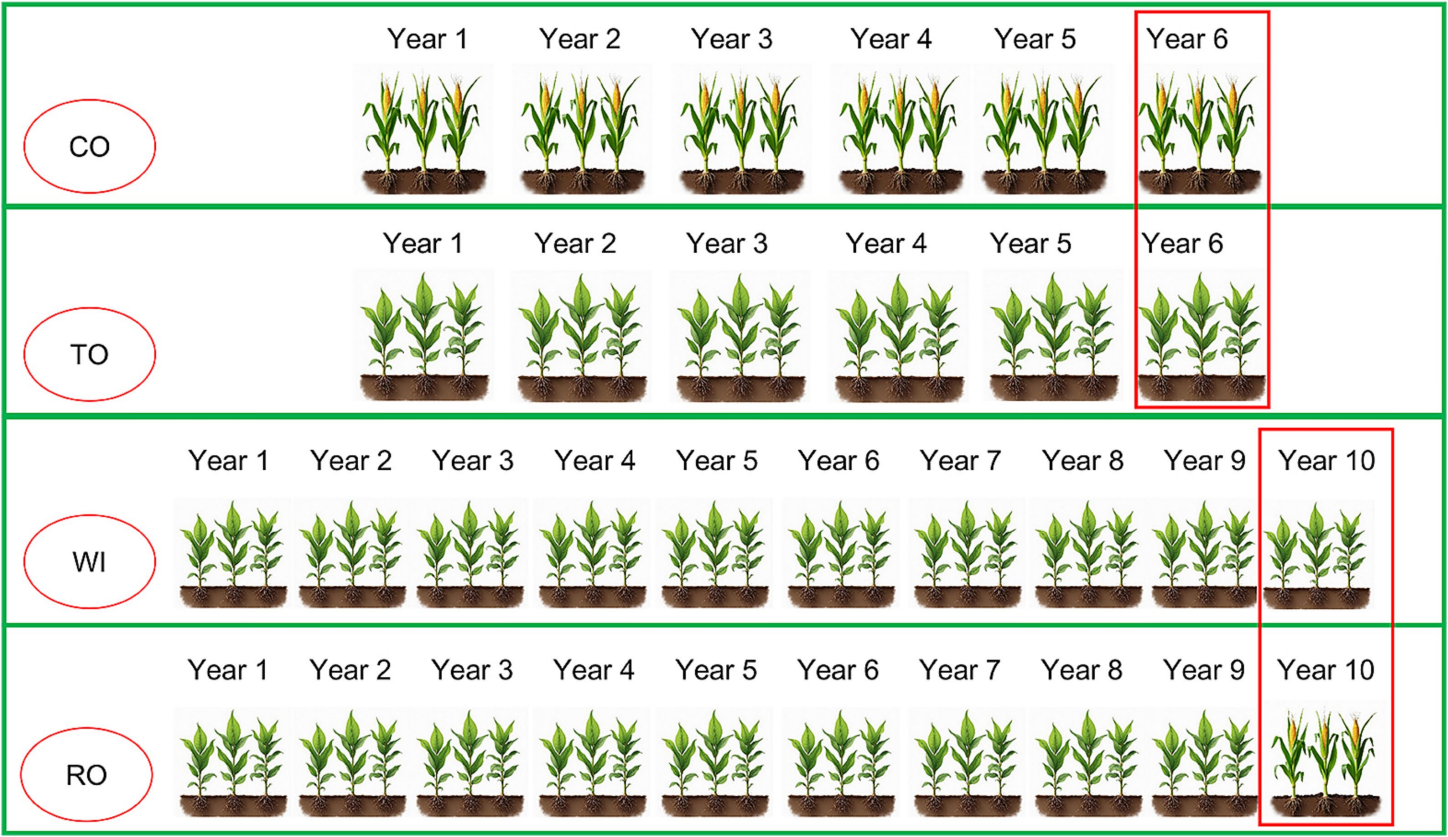
Schematic diagram showing the four cultivation patterns. CO, corn monoculture for 6 years; TO, tobacco monoculture for 6 years; WI, tobacco monoculture for 10 years with serious wilt; RO, tobacco monoculture for 9 years and corn rotation for 1 year. Red borders indicate the collection time of the soil samples.

The experimental site was located at the Malipo long-term continuous cropping experimental station in Wenshan Prefecture, Yunnan Province, China (23°7′43.3″N, 104°42′0.6″E). The zonal soil in this area is predominantly red soil. For sampling, we selected the following four fields as experimental sites: corn monoculture for 6 years (CO), tobacco monoculture for 6 years (TO), tobacco monoculture for 10 years with severe wilt (WI), and tobacco monoculture for 9 years, followed by 1 year of corn rotation (RO) (Figure 1). Field management practices were consistent across all experimental sites. The base fertilizer application rates were as follows: N: 90 kg·ha^−1^, P_2_O_5_: 90 kg·ha^−1^, and K_2_O: 120 kg·ha^−1^. Topdressing was applied twice, every 30 d, at rates of N: 25 kg·ha^−1^, P_2_O_5_: 25 kg·ha^−1^, and K_2_O: 50 kg·ha^−1^.

### Soil collection and survey of the main diseases in the field experiment

Samples were collected in June 2023, marking the 10th year of the long-term experiment. The samples were collected from the four fields on the same day. Plants from each row (15 plants total per plot) were sampled using a hand trowel. Soil closely attached to the roots was collected as rhizosphere soil. Five soil samples were combined to form a single sample for each treatment. All soil samples were divided into two portions. One portion was stored at −80°C for DNA extraction, and the other was used to analyze soil physicochemical properties. The soil physicochemical properties, including soil pH, soil organic carbon (SOC), available phosphorus (AP), available potassium (AK), and alkali-hydrolyzed nitrogen (AN), were determined using previously described methods (Bao, 2005; Ali et al., 2021). The soil properties of the field experiment are shown in Table 1.

**Table 1:** Soil properties of the field experiment.

Samples	SOC (g⋅kg^−1^)	AN (mg·kg^−1^)	AP (mg·kg^−1^)	AK (mg·kg^−1^)	pH
CO	30.67 ± 0.43 a	218.98 ± 1.00 a	27.51 ± 0.15 d	400.79 ± 0.25 a	5.83 ± 0.03 a
TO	29.52 ± 0.23 ab	102.13 ± 0.71 d	32.36 ± 0.40 c	324.94 ± 0.63 b	5.35 ± 0.03 b
WI	27.11 ± 0.19 c	133.55 ± 0.27 c	86.81 ± 0.27 b	222.13 ± 0.27 c	4.28 ± 0.10 d
RO	28.49 ± 0.93 bc	191.04 ± 0.28 b	97.37 ± 0.27 a	127.86 ± 0.30 d	4.96 ± 0.01 c

SOC, soil organic carbon; AN, alkali-hydrolyzed nitrogen; AP, available phosphorus; AK, available potassium. Different letters in the same column indicate a significant difference among all treatments tested by one-way ANOVA (*p* < 0.05). CO, corn monoculture for 6 years; TO, tobacco monoculture for 6 years; WI, tobacco monoculture for 10 years with serious wilt; RO, tobacco monoculture for 9 years and corn rotation for 1 year.

Based on the observation of typical wilt symptoms (including necrosis and leaf drooping) in corn and tobacco, a bioassay of disease incidence was conducted at 100 days. According to the methods described in “Diagnosis and Control of Maize Diseases” (Chen, 1999) and “General Administration of Quality Supervision, Inspection and Quarantine of the People’s Republic of China, Tobacco Pest and Disease Classification and Survey Methods (GB/T 23222) (Beijing: Standardization Administration, 2008)”, the disease rate was assessed by evaluating the presence of typical wilt symptoms.

### Analysis of the soil microbial communities

Total DNA was extracted from all soil samples using a FastDNA^®^ SPIN Kit according to the manufacturer’s instructions. The V3–V4 regions of the16S rRNA genes were amplified using the bacterial primers 338F (5′-ACTCCTACGGGAGGCAGCAG-3′) and 806R (5′-GGACTACHVGGGTWTCTAAT-3′) (Zhang M. M. et al., 2023). The universal primers ITS1F (5′-CTTGGTCATTTAGAGGAAG TAA-3′) and ITS2R (5′-GCTG CGTTCTTCATCGATGC-3′) were used to amplify the fungal ITS1 region (Jin et al., 2022). The PCR products were checked using 2% gel electrophoresis and subsequently sent to Majorbio Co., Ltd. (Shanghai, China) for paired-end sequencing on the Illumina PE300 platform. After merging and quality checking, high-quality sequences were clustered into operational taxonomic units (OTUs) based on a 97% similarity threshold using UPARSE (Edgar, 2013; Bolyen et al., 2019). Bacterial and fungal taxonomies were assessed against the 16S rRNA database (Silva v138) and the fungal ITS database (UNITE v7.2), respectively (Liu et al., 2021c).

### Statistical analysis

All statistical analyses and data presentation were conducted using R software (v4.3.2). One-way analysis of variance (ANOVA) was performed to evaluate the effects of the cultivation patterns on the soil properties. Alpha and beta diversity were calculated using QIIME (v 1.9.1). Principal coordinate analysis (PCoA), redundancy analysis (RDA), and Spearman’s rank correlation heatmap analysis were used to reveal the connection between the soil physicochemical properties and rhizosphere microbiota. The relative importance of each environmental factor in independently accounting for the total variation was quantified using the hierarchy algorithm, and the results were drawn using the “rdacca.hp” package. The Mantel test was conducted to identify the main determinants of the core microbiome in the soil. Stacked histograms showing the functional abundance of the rhizosphere microbiota were created using the ggplot2 R package. Other analyses were conducted on the Majorbio Cloud platform^1^ using various R packages and workflow frameworks.

## Results

### The disease incidence and disease index in the field experiment

The most prevalent disease affecting tobacco was bacterial wilt, while stem rot was predominant in corn at the experimental site (Supplementary Table S1). The bacterial wilt disease incidence and index of WI were significantly (*p* < 0.05) higher than those of TO. Compared to CO, the disease incidence and index of RO were decreased by 63.19 and 61.34%, respectively. The results indicated that long-term continuous cropping increased the incidence and severity of soil-borne diseases, while crop rotation more effectively reduced the incidence and severity of these diseases.

### Overall microbial community diversity

**Figure 2:**
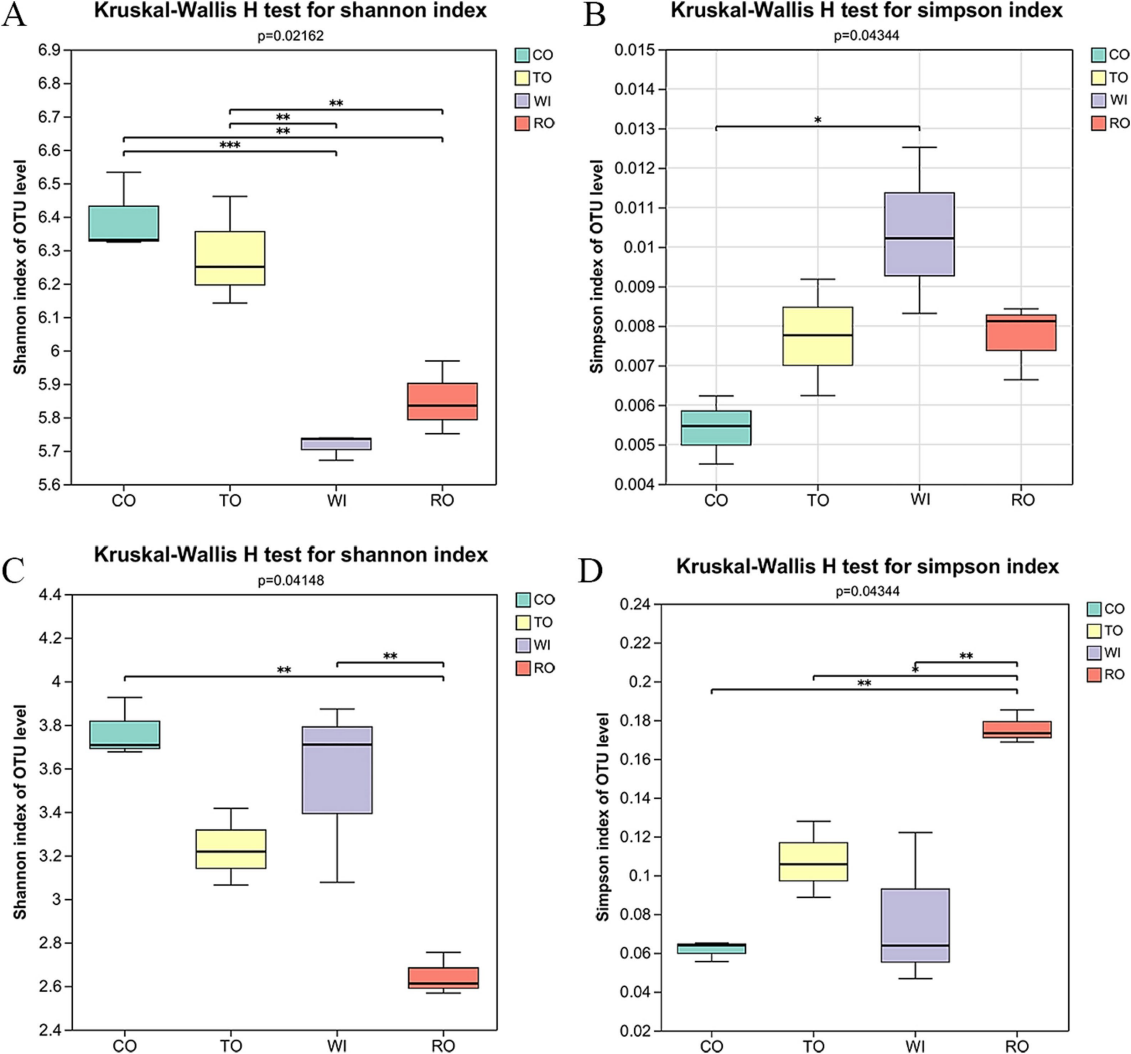
Alpha diversity of the bacteria and fungi in the soil. The Shannon index of the **(A)** bacteria and **(C)** fungi. The Simpson diversity index of the **(B)** bacteria and **(D)** fungi. CO, corn monoculture for 6 years; TO, tobacco monoculture for 6 years; WI, tobacco monoculture for 10 years with serious wilt; RO, tobacco monoculture for 9 years and corn rotation for 1 year. * indicate significant differences among the cultivation patterns. *p < 0.05, **p < 0.01, and ***p < 0.001.

A total of 839,710 high-quality 16S rRNA sequences and 1,047,109 ITS sequences were obtained. At the OTU level, plant pathogenesis was correlated with the diversity of both bacterial and fungal communities. For the bacteria, the soil community diversity in CO (Shannon = 6.396) was higher than that in TO (Shannon = 6.284). However, there were no significant differences between WI and RO (Figure 2A). WI resulted in a significant increase in the number of fungal community OTUs (Shannon = 3.553) compared to RO (Shannon = 2.645) (Figure 2C). The long-term monoculture decreased the soil bacterial and fungal diversity, while the rotations of corn and tobacco increased the rhizosphere microbiota diversity (Figures 2B, D).

### Soil microbial community distribution

**Figure 3:**
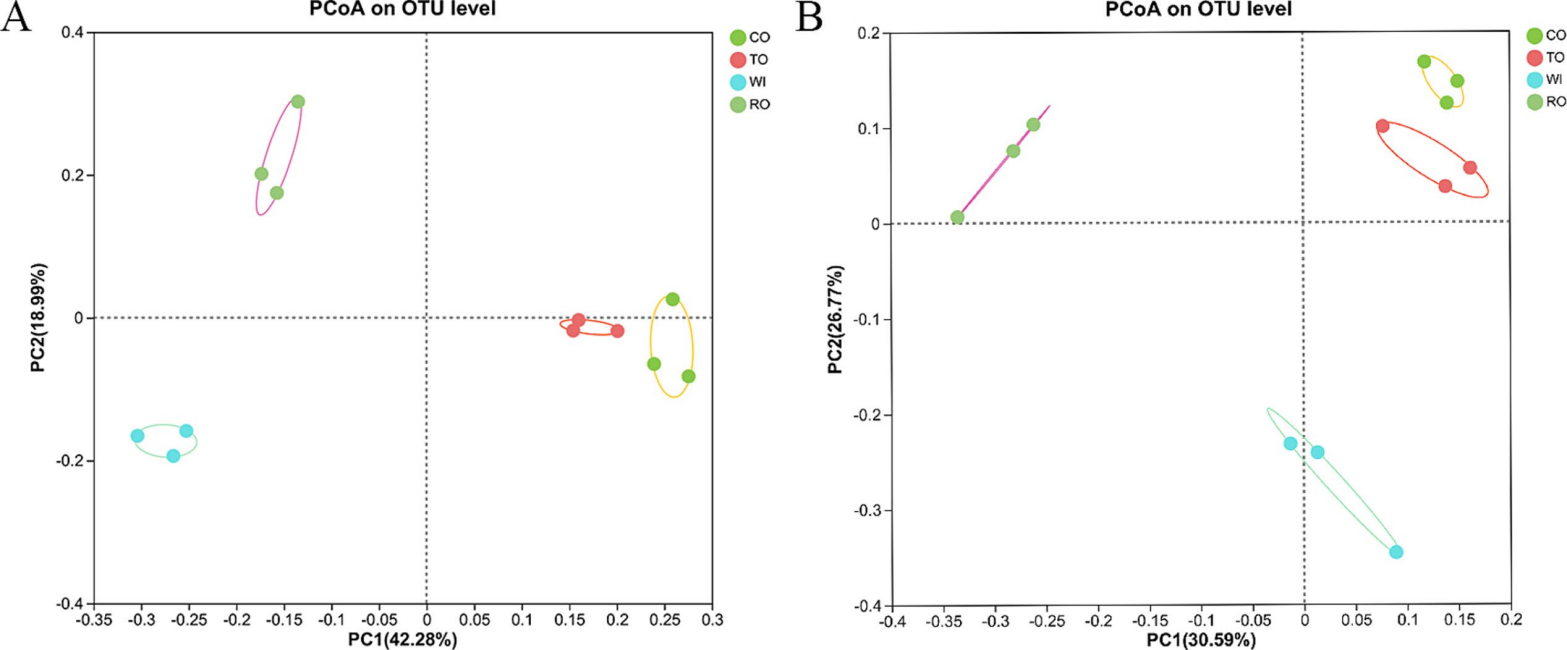
Principal coordinate analysis (PCoA) plots of the soil **(A)** bacterial and **(B)** fungal community structures based on the Bray–Curtis distance.

Principal coordinate analysis based on the Bray–Curtis distance was used to analyze the rhizosphere community structure (Figure 3). For the bacteria, the first principal component (PC1) and the second principal component (PC2) contributed 61.27% of the variance, indicating that they effectively represented the characteristics of the bacterial community composition. The groups CO and TO were clustered together, indicating that their bacterial community compositions were similar but differed significantly from the other groups. The PCoA assigned the fungal communities into three groups: WI, RO, and CO/TO. There was a noticeable distance between WI and RO. The first two axes accounted for 30.59 and 26.77% of the total variability, respectively. PERMANOVA revealed that the rotations of corn and tobacco significantly affected the bacterial (*F* = 5.20, *p* = 0.001) and fungal (*F* = 14.63, *p* = 0.001) community structures.

### Soil microbial community composition

**Figure 4:**
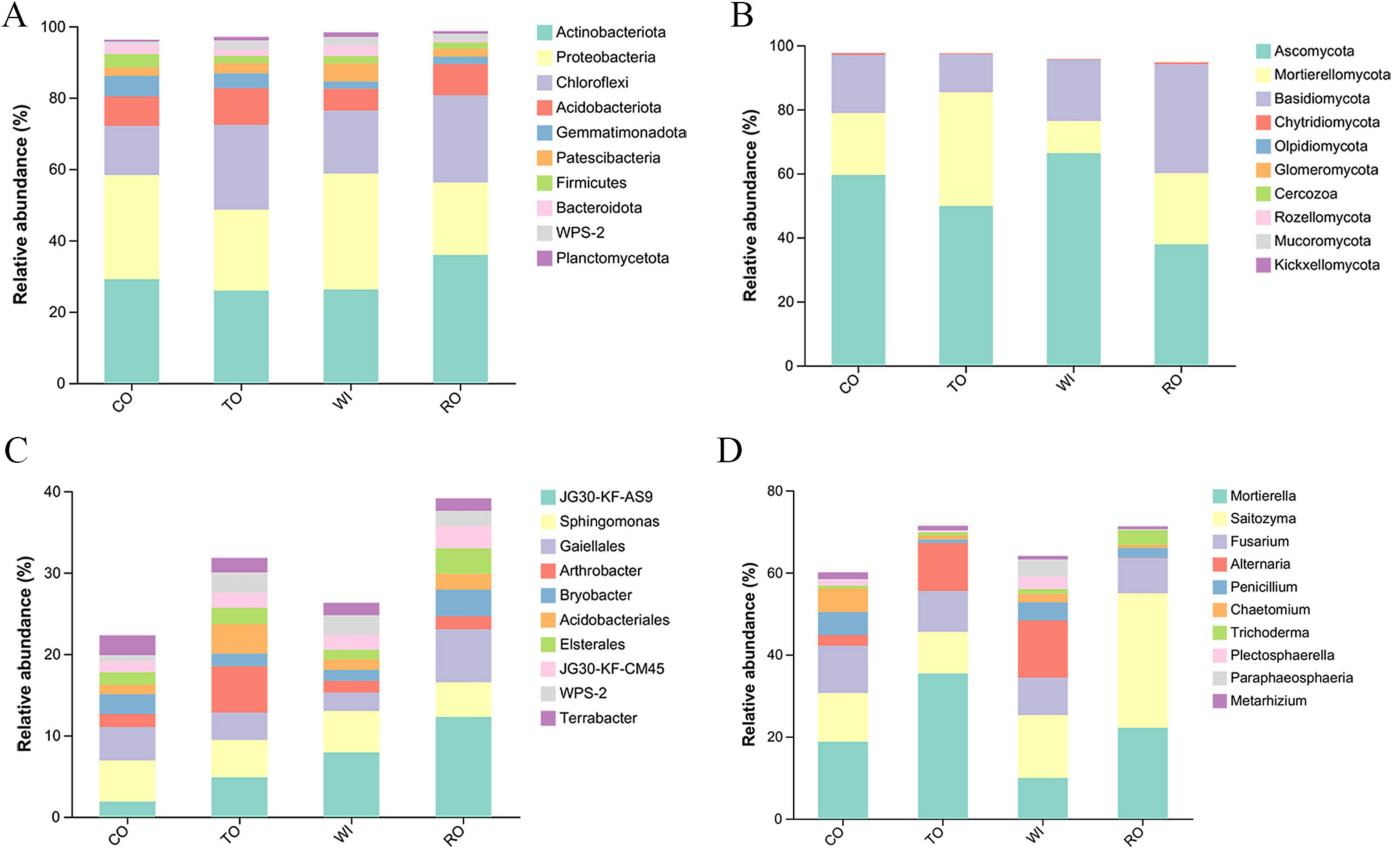
Bacterial and fungal community structures under different cultivation patterns. Bacterial **(A)** and fungal **(B)** community structures at the phyla level. Bacterial **(C)** and fungal **(D)** community structures at the genus level. CO, corn monoculture for 6 years; TO, tobacco monoculture for 6 years; WI, tobacco monoculture for 10 years with serious wilt; RO, tobacco monoculture for 9 years and corn rotation for 1 year.

A total of 46 bacterial phyla were detected across all soil samples. Among these, Actinobacteria, Proteobacteria, and Chloroflexi were the most abundant phyla in the four groups, with Actinobacteria (35.95%) being particularly dominant in the RO group (Figure 4A). The 10 most abundant genera were *JG30-KF-AS9*, *Sphingomonas*, *Gaiella*, *Arthrobacter*, *Bryobacter*, *Acidobacterium*, *Elsterales*, *JG30-KF-CM45*, *WPS-2*, and *Terrabacter* (Figure 4C). The relative abundance of *JG30-KF-AS9*, *Arthrobacter*, and *Acidobacterium* in TO was higher than that in CO. In RO, the relative abundance of *JG30-KF-AS9*, *Gaiella*, *Arthrobacter*, *Bryobacter*, and *Arthrobacter* decreased to 7.93, 2.24, 1.44, 1.33, and 1.31%, respectively.

Basidiomycota and Ascomycota were the most abundant fungal phyla across all samples. The 10 most abundant genera were *Mortierella*, *Saitozyma*, *Fusarium*, *Alternaria*, *Penicillium*, *Trichoderma*, *Plectosphaerella*, *Paraphaeosphaeria*, and *Metarhizium* (Figure 4B). *Mortierella* was more abundant in TO (35.43%) (Figure 4D). The relative abundance of *Saitozyma* (32.74%) and *Trichoderma* (3.62%) in RO was higher than that in WI. *Alternaria* (13.92%) was more abundant in WI.

To find differences in the bacterial and fungal communities of the soil samples, LEfSE was employed to identify discriminatory biomarkers (LDA scores >3.8) (Supplementary Figure S1). For the bacteria, two phyla—Gemmatimonadota and Myxococcota—and four genera—*Roseiflexus*, *Gemmatimonas*, *Massilia*, and *TK10*—showed higher relative abundance in CO. In contrast, three genera—*Ktedonobacter*, *Vicinamibacter*, and *AD3—*had higher relative abundance in TO. The genera enriched in RO included *Gaiella*, *Acidothermus*, *Elsterales*, *Bryobacter*, and *Streptomyces*. In contrast, the genera enriched in the WI sample included members from three phyla—Proteobacteria, Patescibacteria, and Bacteroidota and seven genera, namely *Rhodanobacter*, *Burkholderia*, *Leifsonia*, *LWQ8*, *Modestobacter*, *Geodermatophilus*, and *Chujaibacter*. For the fungi, five genera—*Penicillium*, *Chaetomium*, *Coniophora*, *Clonostachys*, and *Aspergillus*—showed higher relative abundance in CO, while the phylum Mortierellomycota and the genus *Mortierella* showed higher relative abundance in TO. The genera enriched in RO included the phylum Basidiomycota and genera *Saitozyma* and *Conlarium*. The genera enriched in WI included the phylum Ascomycota and genera *Alternaria*, *Paraphaeosphaeria*, *Plectosphaerella*, *Colletotrichum*, and *Arthrobotrys*.

### Analysis of the differential core OTUs in the soil

**Figure 5:**
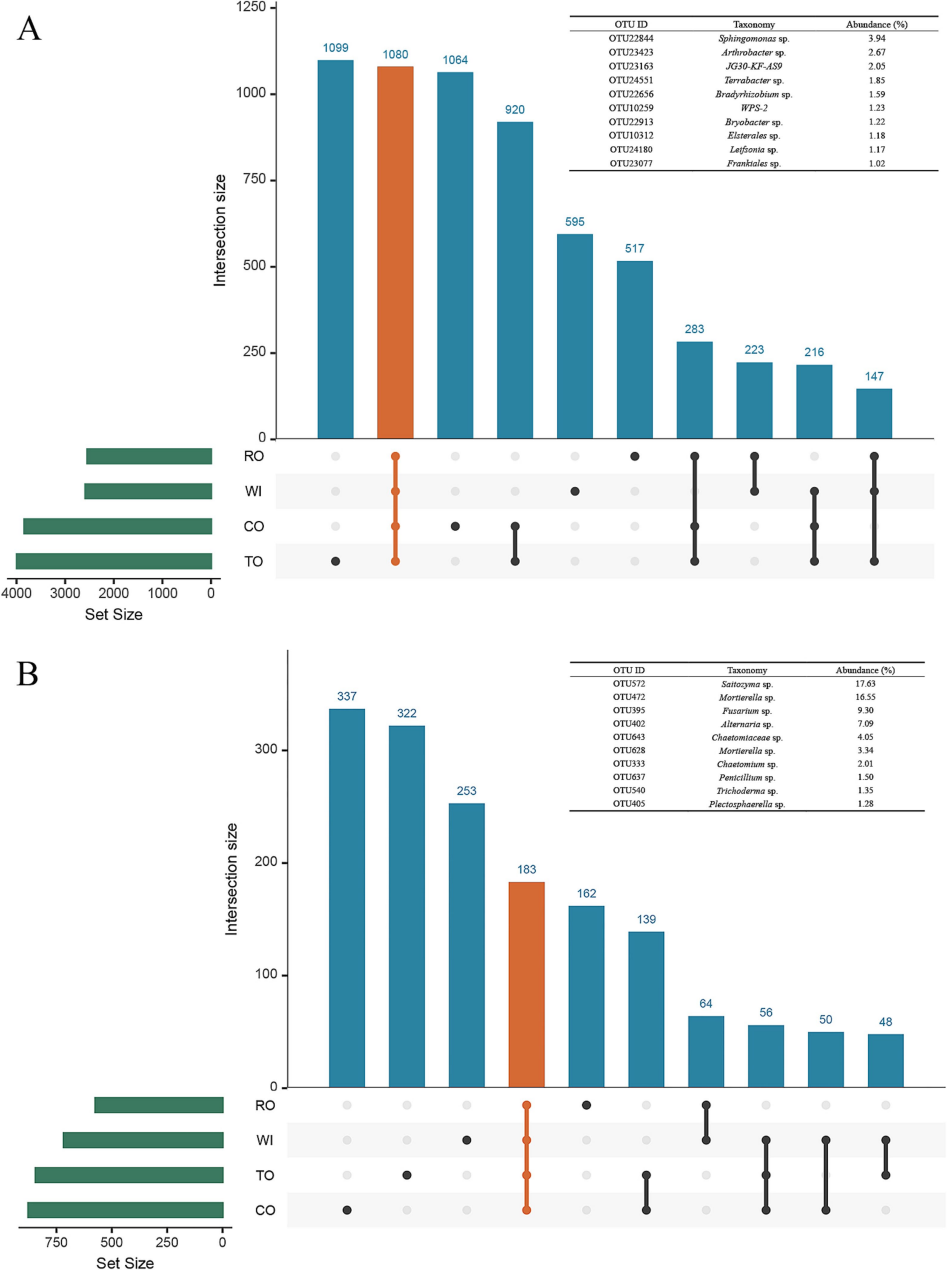
Taxonomic composition of the core bacterial **(A)** and fungal **(B)** microbiomes in the soil. Black dots indicate the presence of the OTUs in the Upset diagram, gray dots indicate the OTUs that were absent, and the lines between different black dots indicate where the OTUs were shared. The OTUs that were common to all five categorical groups (the core microbiome) are represented by orange dots and lines.

For the bacterial communities, Venn analysis revealed that 1,080 OTUs, representing the core microbiome, were shared among the four groups, accounting for 7% of the total sample (Figure 5A). *Sphingomonas*, *Arthrobacter*, *JG30-KF-AS9*, *Terrabacter*, *Bradyrhizobium*, *WPS-2*, *Bryobacter*, *Elsterales*, *Leifsonia*, and *Frankiales* were the top 10 core bacterial genera in the four groups. Among the 10 OTUs, the dominant core taxon was OTU22844 *Sphingomonas* sp., with a relative abundance of 3.94% of the total sample. For the fungal community, a total of 183 OTUs were identified in the four groups, accounting for 18% of the total sample (Figure 5B). *Saitozyma*, *Mortierella*, *Fusarium*, *Alternaria*, *Chaetomiaceae*, *Chaetomium, Penicillium*, *Trichoderma*, and *Plectosphaerella* were the top 10 core fungal genera. The dominant core taxon was OTU572 *Saitozyma* sp., with a relative abundance of 17.63% of the total sample.

### Relationship between environmental factors and microbial communities

**Figure 6:**
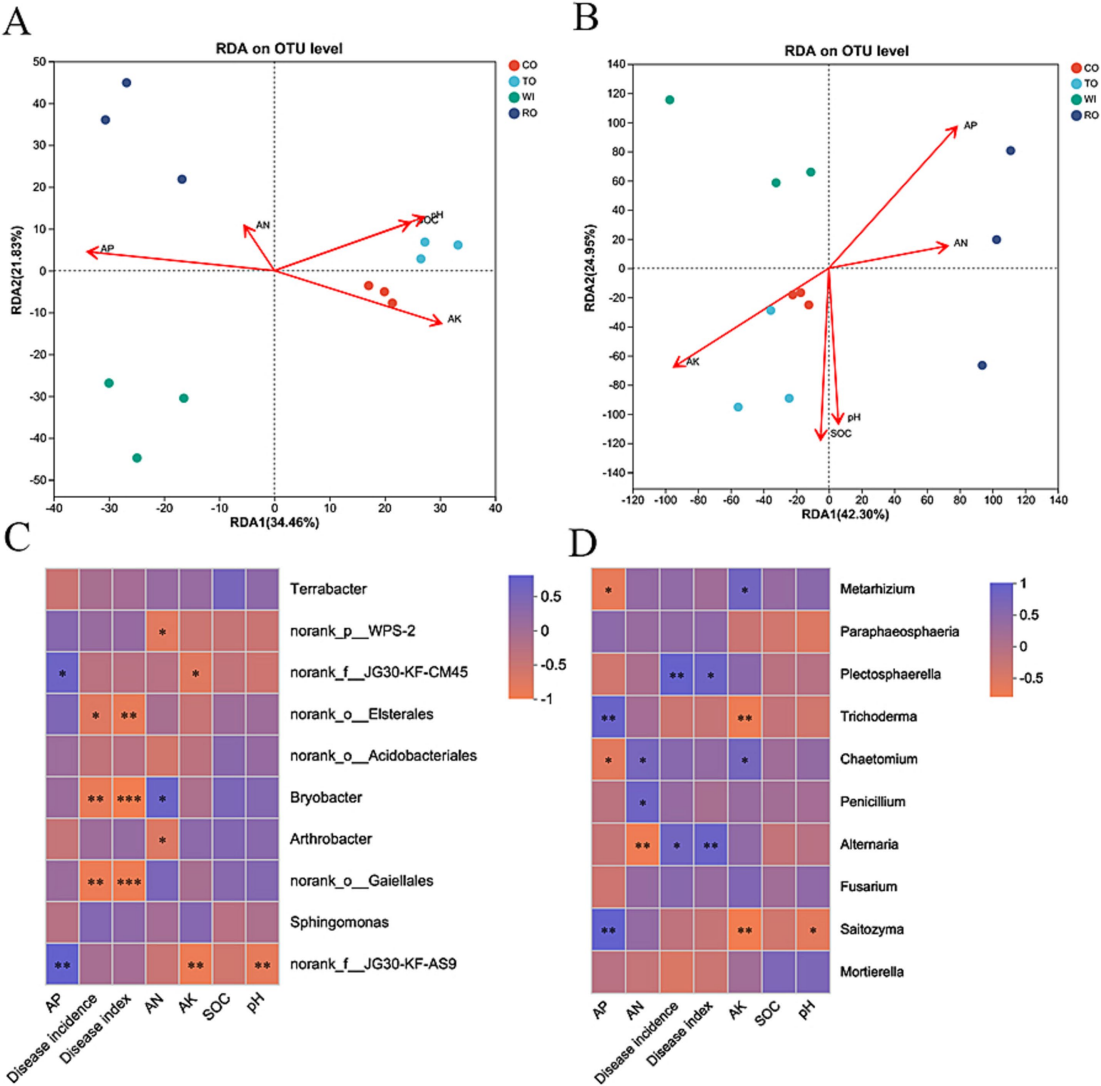
Redundancy analysis (RDA) based on the bacterial **(A)** and fungal **(B)** OTU data with the chemical parameters in the soils. A correlation heatmap of the top 10 bacterial **(C)** and fungal **(D)** genera with the environmental factors. The *R* values are indicated on the right side of the legend with different colors. *p < 0.05, **p < 0.01, and ***p < 0.001.

The relative importance of each explanatory variable in independently accounting for the total variation was quantified using the hierarchy algorithm (Supplementary Figure S2). AP had the greatest effect on the bacterial communities (24.43%), while AK significantly influenced the formation of the fungal communities (24.37%) among the five environmental factors included in this model. In Figure 6A, the first two axes of the RDA explain 34.46 and 21.83% of the total variation in the soil bacterial data, respectively. In Figure 6B, the first two axes of the RDA explain 42.30 and 24.95% of the total variation in the soil fungal data, respectively. We also used Spearman’s rank correlation to evaluate the relationships between the abundant bacterial genera and soil physicochemical properties. The dominant genus *Bryobacter* was positively correlated with AN (*p* < 0.01), while *WPS-2* negatively correlated with AN (*p* < 0.01). For the fungal communities, the dominant genera *Trichoderma* and *Saitozyma* were positively correlated with AP (*p* < 0.01) and negatively correlated with AK and pH (*p* < 0.01). Furthermore, the variation in the core microbiome displayed significant correlations with environmental factors, as evidenced by the Mantel test (*p* < 0.05) (Supplementary Figure S3). We observed that the core bacterial genera were significantly impacted by AK, AP, and AN (*p* < 0.01). AP and AK were also found to impact the core fungal genera (*p* < 0.01). The relative abundance of *Bryobacter* and *Gaiella* was significantly (*p* < 0.001) negatively correlated with the disease index (Figure 6C). The higher abundance of these bacteria in the soil might be helpful for inhibiting bacterial wilt and stem rot. In contrast, the relative abundance of *Plectosphaerella* (*p* < 0.05) and *Alternaria* (*p* < 0.01) were significantly positively correlated with the disease index (Figure 6D). We speculated that these fungi with high relative abundance in the soil may promote the outbreak of soil-borne diseases.

### Network analysis of the soil microbial communities

**Figure 7:**
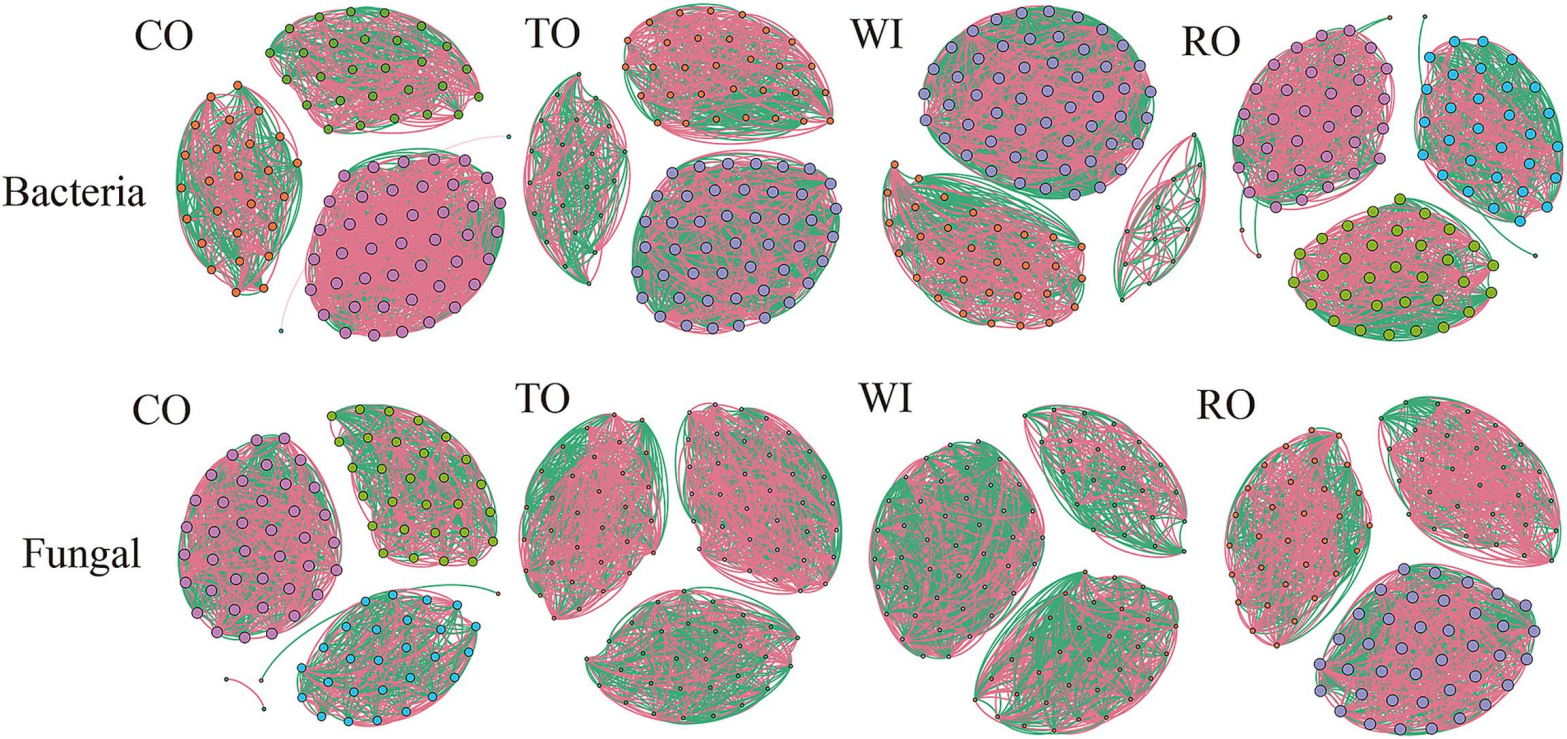
The co-occurrence network of the soil bacteria and fungi. CO, corn monoculture for 6 years; TO, tobacco monoculture for 6 years; WI, tobacco monoculture for 10 years with serious wilt; RO, tobacco monoculture for 9 years and corn rotation for 1 year.

In this study, the differences and interactions of the soil bacterial and fungal communities were confirmed through co-occurrence networks at the OTU level (*R* > 0.7, *p* < 0.05) (Figure 7). For the bacterial communities, the number of edges (1,962) was highest in the soils from RO (Supplementary Table S2). The average degree and network density for the fungal communities were lower in CO compared to RO. Relative to WI, RO increased the average degree and network density values. For the fungal community, the network structure was significantly simpler than that of the bacterial community. The number of edges (1662) was highest in the soils from WI. Furthermore, the number of network edges in CO was fewer compared to all the other groups. Moreover, compared to WI, RO decreased the average degree and network density values. These findings showed that the fungal networks in WI were significantly more complex compared to RO.

### Soil microbial function prediction

**Figure 8:**
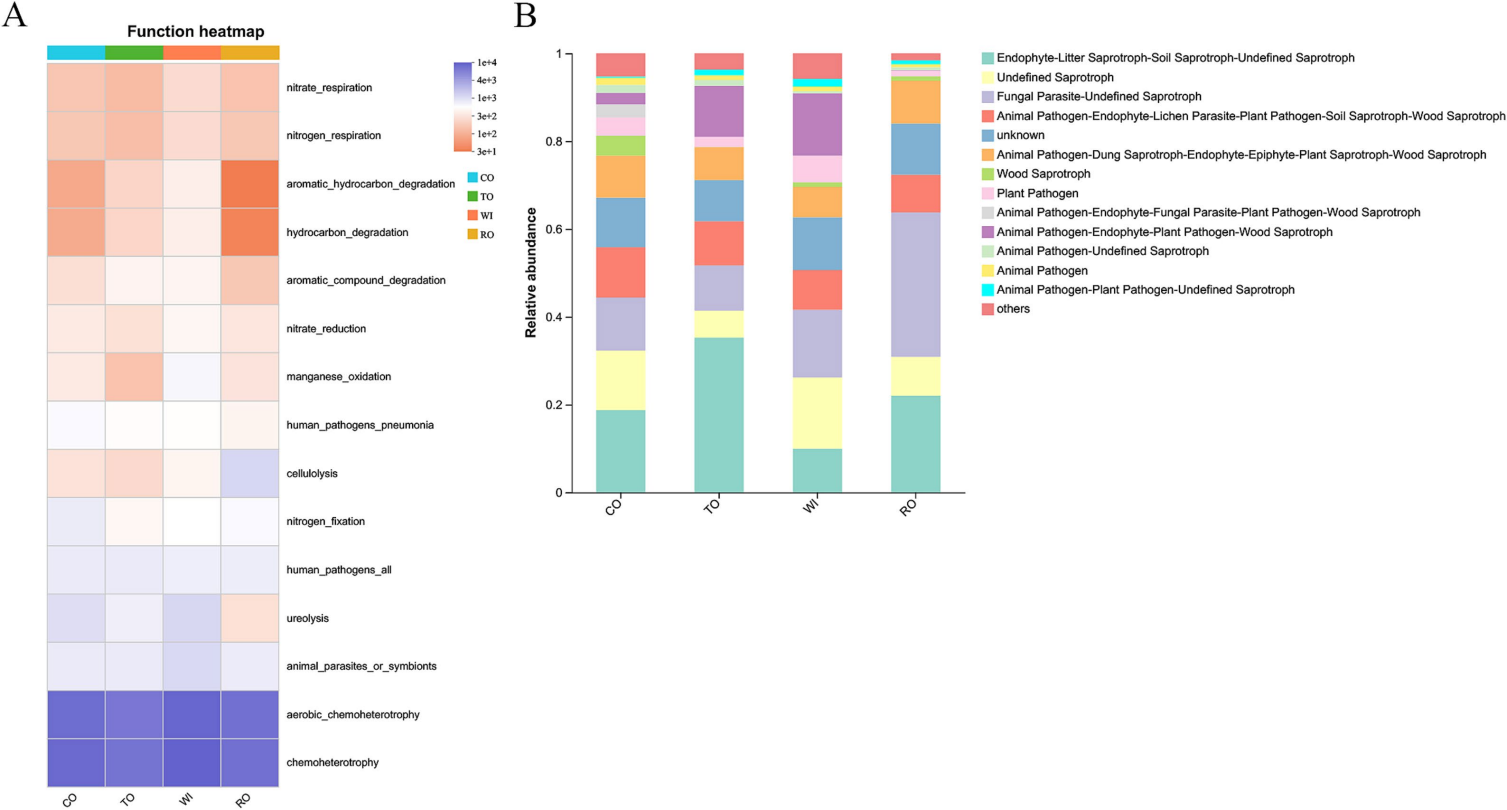
Changes in the functional groups based on the bacterial **(A)** and fungal **(B)** OTU data in the soil. CO, corn monoculture for 6 years; TO, tobacco monoculture for 6 years; WI, tobacco monoculture for 10 years with serious wilt; RO, tobacco monoculture for 9 years and corn rotation for 1 year.

Based on the FAPROTAX database, we predicted the bacterial functions and identified the top 15 functional groups (Figure 8A). RO also showed a decrease in genes related to ureolysis, hydrocarbon_degradation, and aromatic_hydrocarbon_degradation. The FUNGuild database was used to analyze the functional profiles of the fungal communities (Figure 8B). The TO group was dominated by Endophyte-Litter Saprotroph-Soil Saprotroph-Undefined Saprotroph, accounting for 35% of the total community. Animal Pathogen-Endophyte-Plant Pathogen-Wood Saprotroph was dominant in TO and WI, and their relative abundance was 11 and 14%, respectively. The saprotrophs were significantly lower (8%) in the RO group than in the WI group. In addition, animal pathogens and plant pathogens were significantly decreased in the RO group compared to the other groups.

## Discussion

### Microbial community differences among the four cultivation patterns

Continuous cropping is a negative feedback mechanism for soil, which not only changes the soil environment but also promotes the progression of plant diseases (van der Putten et al., 2013). Recent studies have revealed that the stability of microbial community composition is important for a healthy host–microbe relationship and that both enrichment and imbalance in microbiota abundance are important mechanisms for disease development in plants (Schlatter et al., 2017; Berendsen et al., 2018). Our results showed that, compared to continuous tobacco cropping, the corn rotation increased the bacterial diversity. Long-term continuous cropping durations resulted in lower bacterial community diversity (Zhang et al., 2015). The soil bacterial community structure, bacterial species richness, and Shannon index values were significantly increased under crop rotation conditions (Town et al., 2023). Continuous cropping of corn and tobacco increased soil fungal community diversity, which is not completely consistent with previous studies (Zhou et al., 2017; Zhao et al., 2020). These differences may be due to variations in the duration of soil environmental conditions, crop variety, and other factors. We observed that, in the PCoA analysis, the bacterial and fungal communities of CO and TO were clustered, indicating that the soil microbial community structures were affected by the continuous cropping cultivation patterns. The rhizosphere soil microbial communities of TO and CO exhibited a certain degree of homogeneity. In particular, the communities in RO remarkably differed from those in CO, TO, and WI, suggesting that soil bacterial and fungal communities are significantly different between crop rotation and long-term continuous cultivation patterns.

### The influence of the different cultivation patterns on the microbial composition

Crop rotation is an important cultivation practice for crop production and the reduction of pests and diseases. Crop rotation is also considered an important measure for improving soil quality and reducing the use of mineral fertilizers (Câmara-Salim et al., 2021). Crop rotation increased the yield and oil content of canola and decreased disease pressure from *Leptosphaeria* and *Alternaria* (Town et al., 2023). Monocropped cucumber increased soil nutrient concentrations but decreased available nutrient concentrations (Chen et al., 2022). Cotton-grain-rape rotation increased the yield of cotton, maize, and wheat, as well as the above-ground dry matter weight of cotton and maize (Dong et al., 2024). Maize-wheat rotation affected the species composition of *Fusarium*, but no significant difference in pathogenicity was observed between wheat and rice (Dong et al., 2023). This study demonstrated that Actinobacteria, Proteobacteria, and Chloroflexi were the most abundant bacterial phyla in the four groups, with Actinobacteria (35.95%) being more abundant in RO. Similar results have been found in many previous reports (Bulgarelli et al., 2013; Delgado-Baquerizo et al., 2018). Previous studies have shown that Actinobacteria establishes disease suppression through an antagonistic effect (Weller et al., 2002). The imbalance of Actinobacteria in the tomato rhizosphere increases the incidence rate of bacterial wilt (Liu et al., 2021a). In this study, the corn rotation promoted the proliferation of Actinobacteriota. Crop rotation was associated with a more significant effect on soil fungal communities compared to bacterial communities (Kracmarova et al., 2022). The keystone taxa identified in the rotations of rice and canola were all fungal genera (Zhang H. F. et al., 2023). The fungi that were consistently associated with monocropping were known pathogens of tobacco or corn, including *Alternaria* and *Mortierella*. These fungi were enriched in CO, TO, and WI. This finding is in line with previous observations that crop rotation significantly influences the composition of the rhizosphere in canola (Town et al., 2023). The relative abundance of the multiple beneficial fungi, including *Saitozyma* and *Trichoderma*, was increased in the tobacco–corn rotation.

In this study, we focused on several typical microorganisms as these microbial taxa are commonly associated with soil function and crop productivity, directly driving important soil biological processes. At the bacterial genus level, the average relative abundance of some putative biocontrol microbes, such as *Sphingomonas* and *Gaiella*, was significantly promoted by the corn rotation. *Sphingomonas* has been associated with resisting the accumulation of soil-borne pathogens to protect plant health (Lan et al., 2014). It has been reported that *Sphingomonas* could produce carotenoids and improve stress resistance in rice (Cheng et al., 2021). *Gaiella* is a member of the Actinobacteriota phylum and is widely used to control soil-borne plant diseases (Liu et al., 2019). Our results showed that *Sphingomonas* was the core bacterial genus, and *Gaiella was* significantly enriched in the corn rotation. The fungal communities that were significantly enriched in the four groups also differed. *Penicillium* and *Chaetomium*, enriched in CO, have been reported to induce plant resistance to pathogens by activating multiple defense signals (Khalil et al., 2021). *Mortierella* was the core genus enriched in TO, which has been proven to control soil-borne pathogens and improve plant growth (Wani et al., 2017; Mares-Ponce de León et al., 2018). In addition, *Alternaria*, which was consistently associated with the monoculture, being enriched in WI, can cause wilt disease in various crops (Zeng et al., 2024). In addition, *Saitozyma* was enriched in RO, which is well known for its ability to release auxins and lipids (Gorte et al., 2020). Our results suggested that these discriminant biomarkers are critical to soil ecosystem health and induce positive or negative interactions with host plants.

### Soil microbial community response to the environmental factors

Long-term continuous cropping causes the deterioration of soil chemical properties (Ding et al., 2024). Previous studies have shown that pH is one of the most important factors that affect microbial communities in soils (Zhang M. M. et al., 2023). In this study, soil pH was increased in RO and the corn rotation prevented soil acidification. pH had a significant negative impact on the bacteria in the genus *norank_f_JG30-KF-AS9* (*p* < 0.01) and the fungi in the genera *Saitozyma* and *Trichoderma* (*p* < 0.01). This indicates that pH primarily regulates the abundance of multiple beneficial microorganisms in soil. Soil nutrients increased in long-term continuous cropping fields, indicating that the lack of plant nutrients may not directly cause plant diseases (Mbanyele et al., 2022). Our results also revealed that planting corn was more favorable for AN and AP accumulation in the soil. Several reports have found that environmental factors have different effects on soil microorganisms (Dong et al., 2017). Our results showed that the dominant bacterial genus *Bryobacter* was positively correlated with AN, while the dominant fungal genera *Trichoderma* and *Saitozyma* were positively correlated with AP. *Bryobacter* is considered to be a plant growth-promoting rhizosphere bacterium (PGPR) (Vasconcellos et al., 2021). *Bryobacter* improved the diversity and stability of the bacterial community in the rhizosphere soil of tomato, enhancing resistance to *Ralstonia solanacearum* (Zhang J. et al., 2023). *Trichoderma* enhances the absorption of P by plants and strengthens their ability to resist adversity stress (Bononi et al., 2020). *Saitozyma* plays a key role in promoting soil P transformation and accumulation (Li et al., 2022).

### The evolutionary trend of the soil microbial communities

In the co-occurrence analysis, the corn rotation increased the complexity of the bacterial co-occurrence network and decreased the complexity of the fungal co-occurrence network, as indicated by the increased number of edges in the bacterial communities and the reduced average degree and network density in the fungal communities. Previous studies have shown that crop rotation improves the soil microenvironment, allowing more microorganisms to survive freely and reducing cooperation and competition (Fan et al., 2017). We observed that the ecological functional genes related to ureolysis, hydrocarbon_degradation, and aromatic_hydrocarbon_degradation were also decreased in RO, suggesting that accelerated organism decomposition, hydrocarbon, and aromatic_hydrocarbon degradation were facilitated by the improved soil properties. We found that the abundance of the animal pathogens and plant pathogens in TO and WI was higher than that in RO, which may be caused by the interactions between plant pathogens and free microorganisms. Previous studies have shown that plant pathogens secrete enzymes to inhibit the nitrogen restriction of free microorganisms, thus inhibiting organic matter decomposition (Averill, 2016). Therefore, corn rotation in continuous cropping tobacco fields could change plant–soil microbial community composition and has the potential for controlling soil-borne diseases.

Despite the robust design of our study, there are some limitations that should be taken into consideration. Each soil sample represented 15 rhizosphere soils from the plants in each plot, but it did not capture the overall temporospatial profile of the soils affected by serious bacterial wilt. The geographical position also contributed to the differences in the microbial composition over time. Notwithstanding these limitations, the results were clear, supported by a comprehensive analysis of the response of the soil properties and microbial communities under crop rotation. Future research should focus on exploring the functional responses of soil and plant microbiomes to different cultivation patterns, combining soil metabolome analysis to illustrate changes in the soil microecological environment.

## Conclusion

This study examined the effects of continuous cropping and corn rotation cultivation patterns on soil microbial diversity and community structure in tobacco soil on the Yungui Plateau, where severe bacterial wilt is prevalent. The corn rotation altered the soil bacterial and fungal communities, increased their diversity, and improved the soil microenvironment. Furthermore, corn rotation fostered synergistic increments in the beneficial microorganisms. The cultivation patterns of corn rotation may be more conducive to the sustainable development of the karst agricultural system.

## Data Availability

All sequence data have been deposited in NCBI Sequence Read Archive database under accession number PRJNA1166728.

## Footnotes

^1^ https://cloud.majorbio.com/page/project/p.html
