# Supplementary figures and images for: Understanding the microbiome–crop rotation nexus in karst agricultural systems: insights from Southwestern China
Source: Front Microbiol. 2025 Feb 26;16:1503636. doi: 10.3389/fmicb.2025.1503636 (PMC11897573; doi:10.3389/fmicb.2025.1503636)

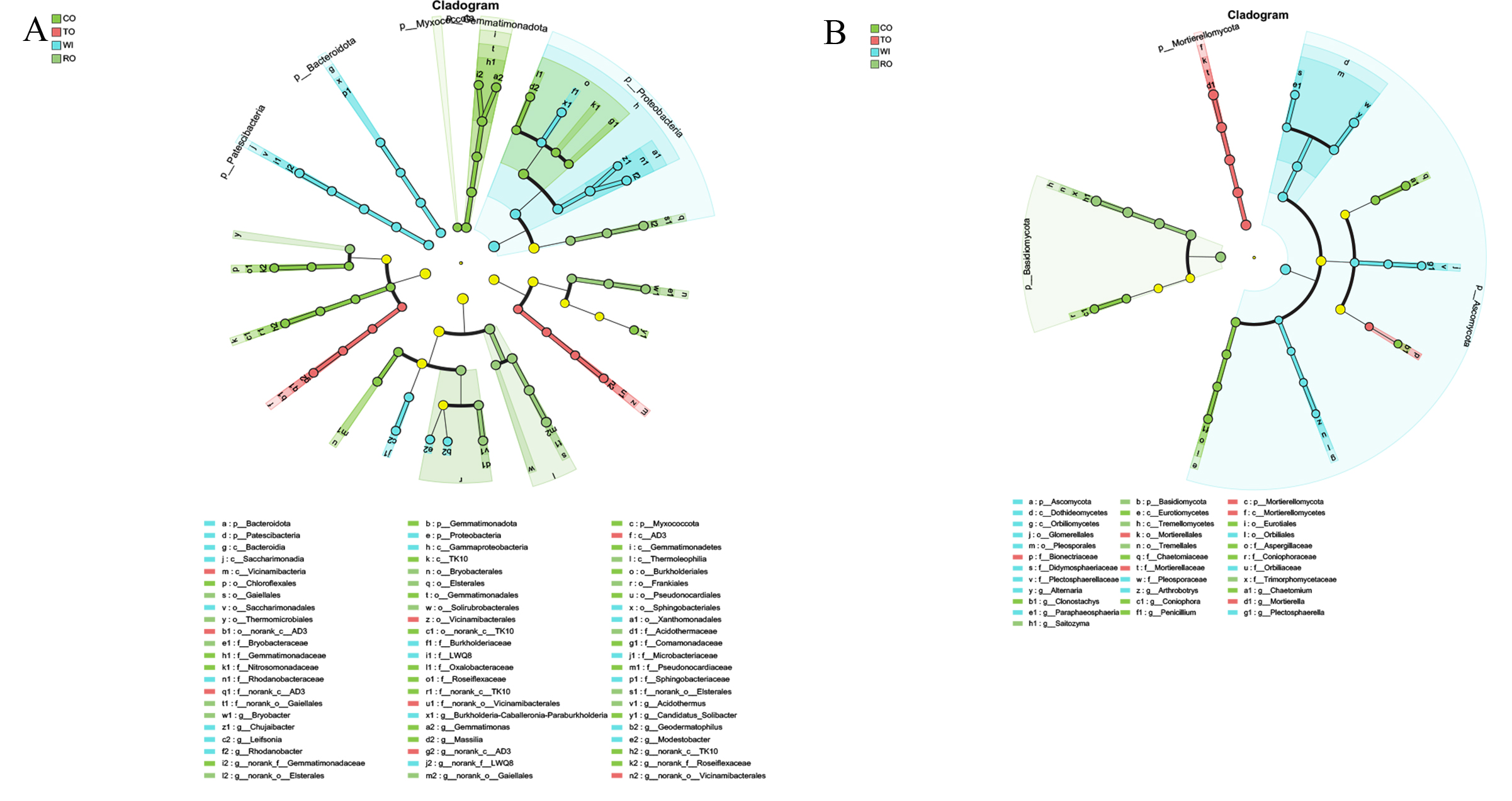

Supplement: Supplementary file 1 [file Image_1.JPEG]

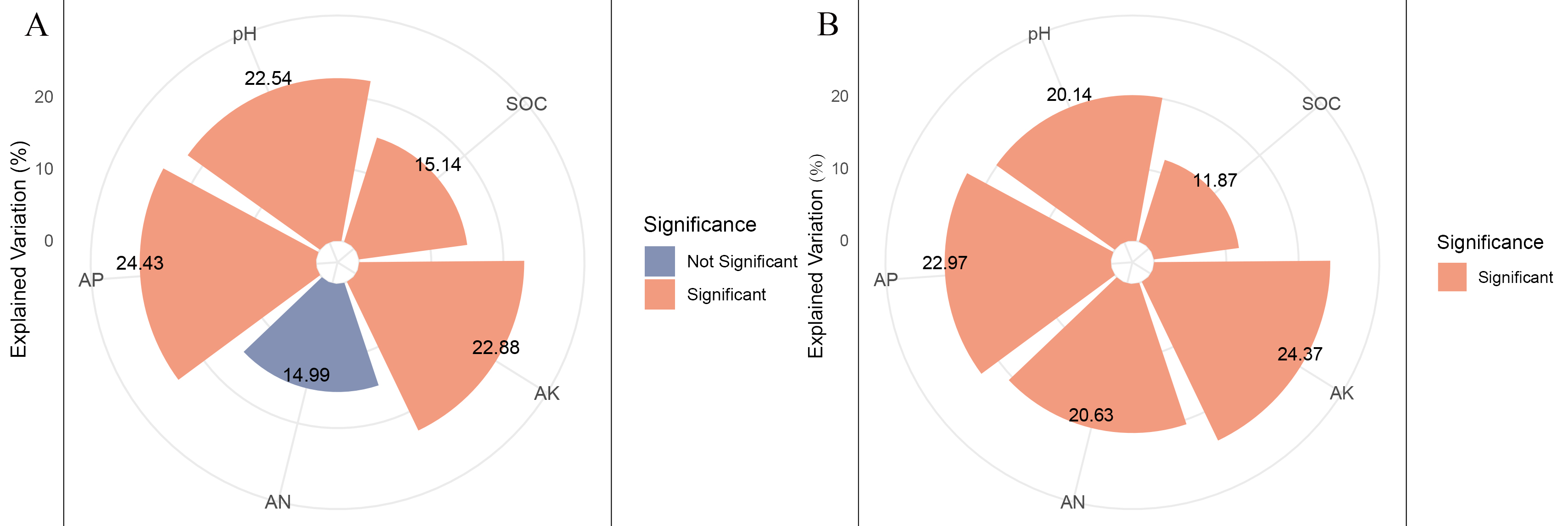

Supplement: Supplementary file 2 [file Image_2.JPEG]

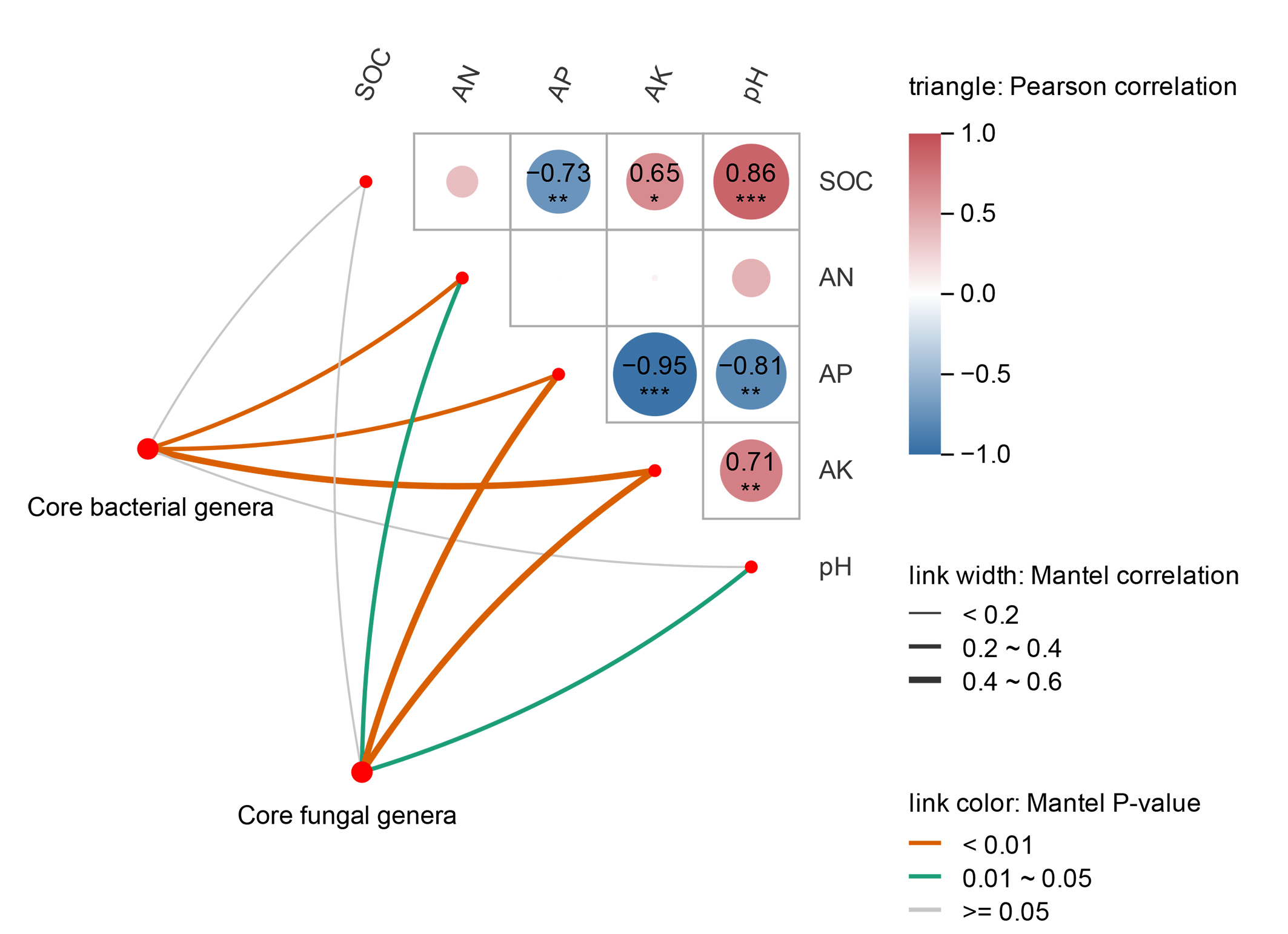

Supplement: Supplementary file 3 [file Image_3.JPEG]
