# Supplementary material for: Understanding the microbiome–crop rotation nexus in karst agricultural systems: insights from Southwestern China
Source: Front Microbiol. 2025 Feb 26;16:1503636. doi: 10.3389/fmicb.2025.1503636 (PMC11897573; doi:10.3389/fmicb.2025.1503636)
Supplement: Supplementary file 4 [file Table_1.docx]

**Supplementary Figure S1.** The LEfSE analysis of differences in bacterial (A) and fungal (B) abundances among different treatments with a threshold value of 3.8. The taxonomic clades map showed the taxonomic rank relationships of the major taxa in the sample community from phylum to genus (from inner circle to outer circle). Node size corresponds to the average relative abundance of the taxon. Nodes with colors indicate that these taxa showed significant differences between groups, and their abundance was higher in the grouped samples represented by this color. Letters identify taxon names that differed significantly between groups.

**Supplementary Figure S2.** The relative importance of each explanatory variable in independently accounting for the total variations in bacterial (A) and fungal (B) communities was quantified by applying the hierarchy algorithm. SOC, Soil organic carbon; AP, Available phosphorus; AK, Available potassium; AN, alkali-hydrolyzed nitrogen.

**Supplementary Figure S3.** Correlation analysis among soil environmental properties and core microbiome based on the Mantel test. The line color indicates the significance level of differences (*P*-values), while the line size represents the correlation coefficients (Mantel's r). Asterisks (*) indicate significance: **P* < 0.05, ***P* < 0.01, ****P* < 0.001.
