# Supplementary material for: Understanding the microbiome–crop rotation nexus in karst agricultural systems: insights from Southwestern China
Source: Front Microbiol. 2025 Feb 26;16:1503636. doi: 10.3389/fmicb.2025.1503636 (PMC11897573; doi:10.3389/fmicb.2025.1503636)
Supplement: Supplementary file 5 [file Table_2.DOCX]

**Supplementary Table S1.** Survey of the mainly diseases in the field experiment.

| Samples | Bacterial wilt disease incidence (%) | Bacterial wilt disease index | Stem rot disease incidence (%) | Stem rot disease index |
| --- | --- | --- | --- | --- |
| CO | - | - | 72.46±6.62a | 22.43±6.16a |
| TO | 75.00±6.21b | 28.34±2.35b | - | - |
| WI | 95.00±7.07a | 56.19±9.72a | - | - |
| RO | - | - | 26.67±6.67b | 8.67±3.98b |

Note: Data are expressed as mean ± standard error (*n* = 3), and different letters in the same column indicate significant differences between treatments (*P* < 0.05).

**Supplementary Table S2.** Co‑occurrence networks in the bacterial and fungal communities under different cultivation patterns.

|  | Bacteria | | | | Fungi | | | |
| --- | --- | --- | --- | --- | --- | --- | --- | --- |
|  | CO | TO | WI | RO | CO | TO | WI | RO |
| Edges | 1607 | 1777 | 1463 | 1962 | 1500 | 1561 | 1662 | 1612 |
| Modules | 4 | 3 | 5 | 3 | 5 | 3 | 3 | 3 |
| Average degree | 32.46 | 17.77 | 14.63 | 40.45 | 30.303 | 31.85 | 33.91 | 32.56 |
| Network density | 0.331 | 0.359 | 0.296 | 0.421 | 0.309 | 0.328 | 0.350 | 0.332 |
